# Antibacterial and Antibiofilm Efficacy of Copper-Doped Phosphate Glass on Pathogenic Bacteria

**DOI:** 10.3390/molecules28073179

**Published:** 2023-04-03

**Authors:** Sunaina Shetty, Priyadharshini Sekar, Raghavendra M. Shetty, Ensanya Ali Abou Neel

**Affiliations:** 1 Preventive and Restorative Dentistry Department, College of Dental Medicine, University of Sharjah, Sharjah P.O. Box 27272, United Arab Emirates; 2 RIMHS, Sharjah Institute for Medical Research, University of Sharjah, Sharjah P.O. Box 27272, United Arab Emirates; 3 Department of Clinical Sciences, College of Dentistry, Ajman University, Ajman P.O. Box 346, United Arab Emirates; 4 Center of Medical and Bio-Allied Health Sciences Research, Ajman University, Ajman P.O. Box 346, United Arab Emirates; 5 Department of Pediatric and Preventive Dentistry, Sharad Pawar Dental College and Hospital, Datta Meghe Institute of Higher Education and Research (Deemed-to-be University), Wardha 442001, India; 6 UCL Eastman Dental Institute, Biomaterials & Tissue Engineering, Royal Free Hospital, Rowland Hill Street, London NW3 2QG, UK

**Keywords:** copper-doped phosphate glass, time-kill assay, antibacterial activity, antibiofilm assay and confocal scanning microscopy

## Abstract

This study aimed to investigate the antibacterial [minimal inhibitory concentration (MIC) and minimal bactericidal concentration (MBC)] and antibiofilm activity [log10 colony forming unit/mL (CFU/mL) and biofilm disruption] of copper-doped phosphate glass (CDPG) against *Streptococcus oralis, Enterococcus faecalis, Lactobacillus casei, Staphylococcus aureus, Escherichia coli,* and *Pseudomonas aeruginosa*. Methods: the antibacterial activity was determined using microbroth dilution and time-kill assay. The antibiofilm activity was investigated using crystal violet and confocal laser scanning microscopy. Bacteria growing in absence of CDPG were used as controls. Results: the MIC was ≥125 mg of CPDG/mL; the log10 CFU/mL reduction ranged from 2.66–3.14 to 6.23–9.65 after 4 and 24 h respectively. Generally, no growth was observed after 24 h of treatment with CDPG; the MBC was 250 mg/mL for *L. casei* and *S. oralis* while 500 mg/mL for the rest of the bacteria. The highest and lowest antibiofilm activity was observed against *S. oralis* and *E. coli* respectively. Three patterns of complete biofilm disruption were seen: (i) large areas with *E. fecalis* and *S. oralis*, (ii) medium-size pockets with *S. aureus* and *P. aeruginosa,* or (iii) small areas with *E. coli* and *L. casei*. Conclusion: CDPG can be potentially used as an antibacterial and an antibiofilm agent against oral biofilm-forming bacteria.

## 1. Introduction

The oral mucosal surfaces constantly encounter an overabundance of microorganisms [1]. Several bacterial strains are commonly present in the oral cavity and responsible for many diseases such as caries, periodontitis, osteomyelitis, endodontic infection, and mucosal infections, which involve both Gram-positive microorganisms (*Enterococcus faecalis, Staphylococcus aureus, Streptococcus oralis,* and *Lactobacillus casei*) and Gram-negative bacteria (*Pseudomonas aeruginosa* and *Escherichia coli*) [2,3,4].

Dental biofilm is an exceedingly complex, multispecies ecosystem where oral microorganisms interact cooperatively or competitively with each other [5]. Biofilm consists of bacteria or fungi that aggregate and aid in the onset and/or the progress of infection. It is therefore considered the major etiological factor for many dental diseases. Biofilm formation is a vital virulence factor of many oral pathogens. It starts with the attachment of primary colonizers (e.g., *Streptococci* and *Actinomyces*) followed by bridging species (e.g., *Fusobacterium nucleatum*) and subsequently by late colonizers (e.g., *Porphyromonas gingivalis* and *Tannerella forsythia*) [6,7]. During this process, complex microbial communities will be formed and become embedded within an extracellular polymeric matrix [8] that constitutes 90% of the biofilm. This matrix confers protection to the pathogenic microorganisms from external antimicrobial agents and also helps to evade the host defense mechanism [9,10]. Although numerous studies focused on developing antimicrobial agents to overcome the virulence factors which render them less effective, most of these efforts are in vain to achieve the desired outcomes. The rapid disintegration of most antimicrobial agents by multiple antibacterial resistance mechanisms is also another challenge presently being faced globally [11,12].

Phosphate-based glasses, a class of the third generation of biomaterials, have recently gained great interest for potential tissue engineering and drug delivery applications [13]. They are degradable and the degradation can be effortlessly controlled by changing the glass chemistry. They can be modified with various metallic oxides that could induce antibacterial [14,15] or remineralizing actions [16]. Examples of these oxides include copper oxide [14], titanium dioxide [15], and gallium oxide [17]. They can also be prepared in a variety of forms as a powder [18], microspheres [15], and fibers [19] to modulate their therapeutic potential. Copper-doped phosphate glasses, for example, are effective as an antibacterial agent against Gram-positive cariogenic cocci (*Streptococcus mutans*), Gram-negative, rod-shaped, opportunistic multidrug-resistant bacteria *Pseudomonas aeruginosa* [18], and Gram-positive skin-related *Staphylococcus epidermidis* [14]. Titanium-doped phosphate glasses have also proven to be biocompatible [20,21] with a promising antibacterial action against *S. mutans* when they were incorporated into total-etch dental adhesives [15]. Furthermore, copper-doped mesoporous glass nanospheres modified experimental dental composites could be a promising path for future investigations of antibacterial properties and ion release [22]. Limited research has been conducted on the antibacterial efficacy of copper-doped phosphate glasses on multiple oral microorganisms and their biofilm-forming capacity, which has led to the research gap in the antibacterial efficacy of copper-doped phosphate glasses on oral monomicrobial and polymicrobial biofilm. This study aimed to investigate the potential use of copper-doped phosphate glass (CDPG) as an antibacterial agent against oral biofilm-forming bacteria without the problem of developing antibiotic-resistant strains. Therefore, the antibacterial and antibiofilm activity of CDPG was tested against typical oral bacteria like *E. fecalis, S. oralis*, and *L. casei* as well as common multidrug-resistant bacterial pathogens like *E. coli, P. aeruginosa,* and *S. aureus*. The tested species involve both Gram-positive and negative microorganisms that are commonly involved in many diseases, as highlighted above. The null hypothesis postulated was that there is no antibacterial and antibiofilm activity of copper-doped phosphate glass (CDPG) against oral biofilm-forming bacteria. If proven successful, this CDPG can be incorporated within various dental materials such as cements, adhesives, and fissure sealants to achieve potent antibacterial functions. It could also serve as an active component in mouth rinses, toothpaste, gels, or any other products to reduce the burden of oral microorganisms.

## 2. Results

### 2.1. Scanning Electron Microscopy

**Figure 1:**
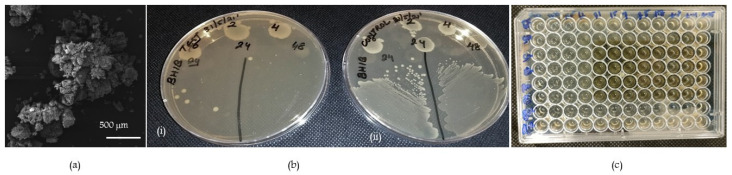
(**a**) Scanning electron microscopy image of copper-doped phosphate glass particles at 100× magnification. (**b**) Representative picture of preliminary test of antibacterial activity (i) Test—*E. faecalis* exposed to 500 mg/mL CDPG at different time points in hours (ii) Control—*E. faecalis* not exposed to 500 mg/mL CDPG. (**c**) Representative image for determination of MIC by broth microdilution method. All six bacterial strains showed a uniform MIC of 125 mg/mL.

As observed in Figure 1a, the glass particles had irregular-shaped morphology and their size varied from 20–100 µm.

### 2.2. Antibacterial Activity

#### 2.2.1. Preliminary Antibacterial Activity and Microbroth Dilution Method

The antibacterial activity of CDPG was assessed against both Gram-positive and Gram-negative bacteria. A preliminary test to check the antibacterial activity of CDPG was performed against all six bacterial strains at different time points in hours. A fixed concentration of 500 mg/mL of CDPG was used for this test. It was observed that the growth of all of the six bacterial strains was completely inhibited by 500 mg/mL of CDPG at 24 h and 48 h. Preliminary testing indicated the antibacterial activity of CDPG. Figure 1b shows a representative picture of the same. Although the bacterial strains could withstand the antibacterial activity of CDPG at 2 h, 4 h, and 6 h, they were completely killed by CDPG at 24 h and 48 h. The MIC of CDPG was determined by the microbroth dilution method. Doubling dilutions of CDPG ranging from 500 mg/mL to 0.25 mg/mL were tested against all six bacterial strains. The minimum concentration of CDPG at which bacterial growth was completely inhibited and visualized with the naked eye, was considered to be the MIC. It was observed that 125 mg/mL of CDPG was the MIC for all the six bacterial strains tested. The uniformity of the results for all of the six bacterial strains tested is shown in Figure 1c. Concentrations of CDPG less than 125 mg/mL did not show any antibacterial activity against all the tested bacterial strains.

#### 2.2.2. Time-Kill Assay

**Figure 2:**
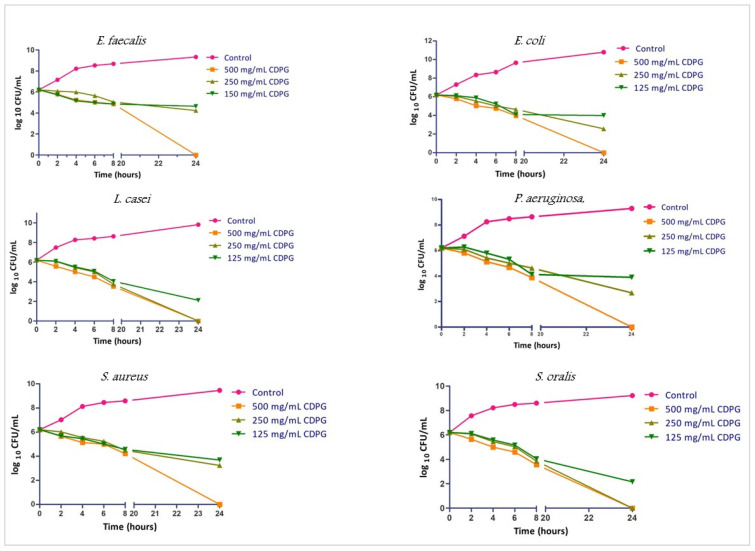
Time Kill assay of different bacteria.

The time-kill assay was performed with 500, 250, and 125 mg/mL CDPG concentrations. Data from the time-kill assays of each microorganism were presented as log10 CFU/mL at different time points, as shown in Figure 2. Generally, the average log10 CFU/mL in the time-kill assay ranged between 5.56 to 5.09 after 4 h of interaction; 4.28 to 4.005 after 8 h of interaction; 3.41 to 0 after 24 h of interaction among the three concentrations of CDPG. All six strains of viable bacteria were completely killed by CDPG at 24 h at 500 mg/mL. It was observed, however, that the common oral microbes—*L. casei* and *S. oralis*—were completely killed by CDPG at 24 h at a MIC of 250 mg/mL as well.

### 2.3. Antibiofilm Activity

**Figure 3:**
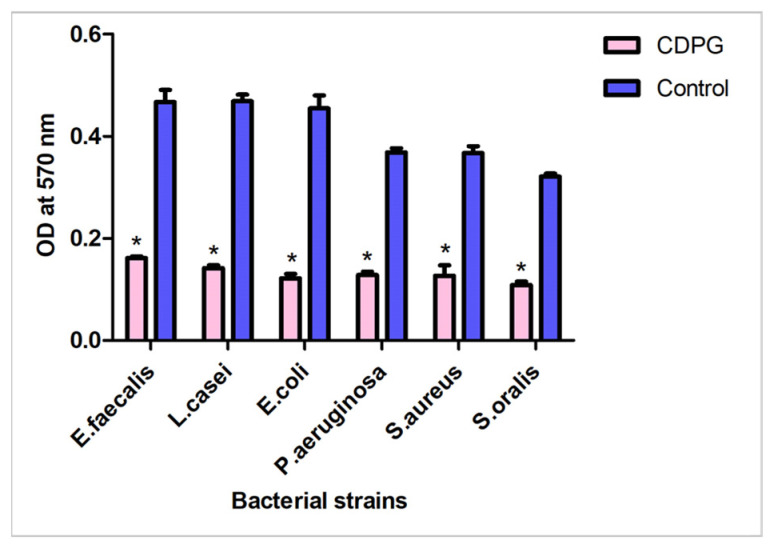
Crystal violet assay for evaluation of the antibiofilm activity of CDPG. * refers to the significant difference from the control with *p* value < 0.05.

A preliminary evaluation of the antibiofilm activity of CDPG was performed by crystal violet staining. The antibiofilm activity was observed with 100 mg/mL of CDPG. The biofilm formed by all the bacteria inoculated with 100 mg/mL of CDPG was significantly lower (*p* < 0.05) than their respective control strain of bacteria grown in the absence of CDPG, see Figure 3. *S. oralis* showed the highest reduction in biofilm with CDPG treatment, while *E. coli* showed the lowest reduction in biofilm with CDPG treatment.

#### Confocal Laser Scanning Microscopy (CLSM) and Disruption of Biofilm

**Figure 4:**
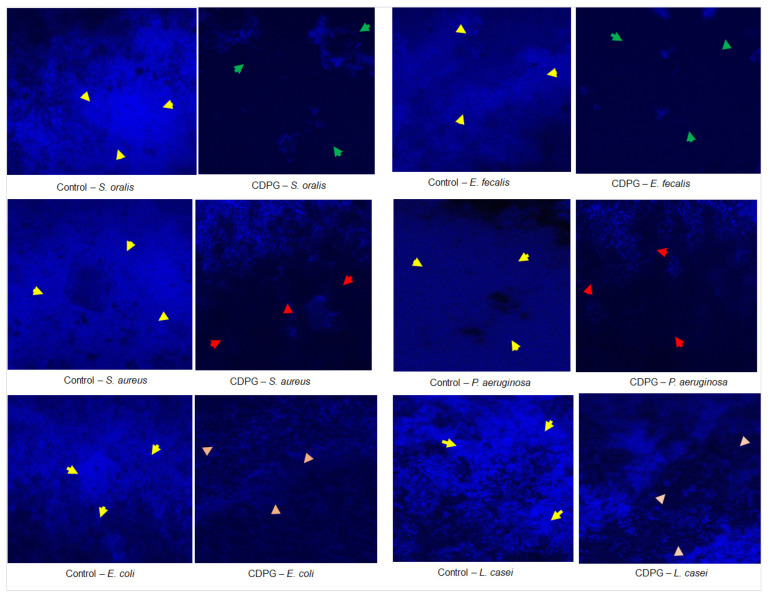
CLSM images showing the disruption of biofilm, produced by different bacterial strains on hydroxyapatite discs, caused by 100 mg/mL CDPG in comparison to the control bacteria growing in the absence of CDPG. Yellow arrows indicate multiple layers of biofilm formed by bacteria. Green arrows indicate large areas of complete disruption of biofilm as seen with *E. fecalis* and *S. oralis*. Red arrows refer to medium-size pockets of complete disruption of biofilm as seen with *S. aureus* and *P. aeruginosa,* while orange arrows refer to small areas of complete disruption of biofilm as seen with *E. coli* and *L. casei*.

The disruption of biofilm, formed by each bacterial strain on hydroxyapatite, by CDPG was visualized by DAPI staining under CLSM. The control samples showed either uniform, thick, or multiple layers of biofilm. On the other hand, the samples treated with 100 mg/mL CDPG for 24 h postbiofilm formation showed one of the three types of disruption of biofilm—(i) large areas of complete disruption of biofilm as observed in *E. fecalis* and *S. oralis*; (ii) medium-size pockets of complete disruption of biofilm as observed in *S. aureus* and *P. aeruginosa,* or (iii) smaller areas of complete disruption of biofilm as observed in *E. coli* and *L. casei* (Figure 4).

## 3. Discussion

This study aimed to investigate the antibacterial and antibiofilm activity of CDPG. In a previous study, the antibacterial activity of CDPG fibers has been tested against *S. epidermidis* and compared with copper oxide-free fibers [11]. The results showed a significant reduction in the number of bacteria attached to CDPG fibers when compared to the control fibers. The higher the copper oxide content, the higher the reduction in the number of attached bacteria. Fibers containing 1 and 5 mol% copper oxides showed 1log10CFU reduction, while those containing 10 mol% showed over 2log10CFU reduction [14]. Similar findings were also observed when the bacteria were grown in the presence of fiber extracts [14]. In another study, CDPG microspheres or microparticles containing 10 mol% copper oxide were used to impart antibacterial action to an experimental phosphate-substituted methacrylate dental adhesive. The results showed that the antibacterial action against *S. mutans* and *P. aeruginosa* varies according to the form of CDPG where CDPG microspheres showed a significantly higher reduction in *P. aeruginosa* than microparticles while the opposite was observed for *S. mutans* [18]. In these studies, the antibacterial action of CDPG was related to the release of copper ions during glass degradation. The amount of copper released varies according to the form (particle, microspheres, or fibers) and the copper oxide content of the glass [14,18]. Regardless of the importance of copper as a cofactor for many enzymatic processes, it is toxic to bacteria by the production of reactive oxygen species (ROS) that cause damage to the bacterial DNA [23].

In the present study, the antibacterial and antibiofilm activity of CDPG was tested against typical oral bacteria like *E. fecalis, S. oralis*, and *L. casei* as well as common multidrug-resistant bacterial pathogens like *E. coli, P. aeruginosa,* and *S. aureus*. It was observed that the MIC of CDPG was 125 mg/mL while the minimum bactericidal concentration (MBC) of CDPG was 500 mg/mL. As highlighted in a previous study, the bactericidal activity was observed with increasing concentrations of copper [24], and with 500 mg/mL of CDPG, more copper ions are expected to be released into the medium than with 125 mg/mL. The time-kill assay indicated that the oral pathobionts *S. oralis* and *L. casei* were completely killed at 24 h with 250 mg/mL, whereas the other bacteria, which are stronger pathogens, required 500 mg/mL to be completely killed by 24 h. The pathogens require a higher concentration of CDPG to be killed when compared to the pathobionts as they inherently carry multiple virulence genes and genes for resistance to chemicals and drugs [25].

Antibiofilm activity was studied by the crystal violet assay. Crystal violet is a dye that belongs to the triphenylmethane dyes family. Crystal violet binds through ionic interactions with bacterial cellular components like DNA [26], proteins [27], and biofilm polysaccharides [28]. The biofilm formation is visualized as a ring around the inner surface of the well after the crystal violet dye is flushed away. Then, this biofilm could be solubilized and measured spectrophotometrically by transferring it to a 96-well plate that has a flat bottom and is optically clear. Since the crystal violet stains the matrix of extracellular DNA, proteins, and polysaccharides, this assay does not correspond directly to the number of viable bacterial cells in the biofilm. The antibiofilm activity was visualized by treating a 24-h bacterial biofilm with CDPG, followed by a crystal violet assay. The spectrophotometric readings of CDPG-treated bacterial biofilms were recorded and compared to untreated bacterial biofilm. The disruption of biofilm varied between the bacteria tested and these results were in concordance with the confocal laser scanning microscopy (CLSM) visualization of the disrupted biofilm on the treatment with CDPG.

As explained above, the antibiofilm activity of CDPG was detected by the crystal violet assay. The antibiofilm activity was observed at a concentration lesser than the antimicrobial concentration (MIC). Results showed that the highest antibiofilm activity of CDPG was observed against *S. oralis* while the lowest activity was observed against *E. coli*. This finding could be related to the nature of both bacteria; *S. oralis* is generally an oral commensal while *E. coli* is a pathogen that causes multiple diseases. Hence, the virulence factors of the pathogen (*E. coli*) are far more developed and thus more difficult to kill when compared to the commensal bacteria as *S. oralis* [29]. *S. oralis* is an oral commensal and opportunistic pathogen. It is well known that *S.oralis* forms a strong biofilm in the presence of mucins from human saliva. *S*. *oralis* is an early colonizer that commences plaque formation by binding to the salivary pellicle on the surface of the tooth [30]. In the present study, the antibiofilm activity was assessed in vitro in the absence of human saliva containing mucins and salivary pellicle. This could be the reason for the CDPG to exhibit the highest antibiofilm activity on *S.oralis*, although it forms an efficient biofilm in the oral cavity.

The disruption of biofilm also varied between bacteria; these results were in concordance with the confocal laser scanning microscopy (CLSM) visualization of the disrupted biofilm on the treatment with CDPG. CLSM detected (i) large areas of complete disruption with *E. fecalis* and *S. oralis*, (ii) medium-size pockets of complete disruption with *S. aureus* and *P. aeruginosa*, and (iii) small areas of complete disruption with *E. coli* and *L. casei*. This could be attributed to the type of biofilm secreted by the respective bacteria, the presence of pili, the amount of glycocalyx present around the bacteria along with the presence of adhesins [31]. The null hypothesis postulated that there was no antibacterial and antibiofilm activity of copper-doped phosphate glass (CDPG) against oral biofilm-forming bacteria. According to the results of this study, CDPG showed significant antibacterial activity against all the tested bacteria as well as a significant reduction in biofilm formation. Therefore, the null hypothesis was rejected.

## 4. Materials and Methods

### 4.1. Glass Preparation

Copper-doped phosphate glass (CDPG), having 50 P_2_O_5_, 30 CaO, 10 Na_2_O, and 10 CuO, was prepared using the melt quenching process [15,16]; the number indicates the mol% of each component. The raw materials used for the glass preparation are phosphorus pentoxide, calcium hydrogen phosphate, sodium dihydrogen phosphate, and copper oxide. The raw materials were mixed and melted in an air furnace (Carbolite, model RHF 1500, UK) at 1100 °C for 2 h. The molten glass was then quenched to room temperature and left to cool overnight. The cooled glass was grounded into a powder using a Retsch PM100 milling machine (Retch GmbH, Germany).

### 4.2. Scanning Electron Microscopy

The glass powder was fixed to a metal stub using a sticky tap and sputter coated with gold-palladium alloy for 120 s using Quorum Technology Mini Sputter Coater, SC7620 under argon atmosphere, 10^−2^ mbar chamber pressure, 18 mA plasma current, and 1 kV applied voltage. The morphology of CDPG was investigated using scanning electron microscopy (Tescan VEGA XM variable pressure SEM).

### 4.3. Antibacterial Activity

For this study, *E. faecalis* (ATCC 219212), *S. aureus* (ATCC 29213), *E. coli* (ATCC 25922), *P. aeruginosa* (ATCC 27853), *S. oralis* (ATCC 6249), and *L. casei* (ATCC 393) were used. These organisms were cultured in brain–heart infusion broth (BHIB, Biolife, Milan, Italy), and the turbidity was matched to 0.5 McFarland for the different assays.

#### 4.3.1. Preliminary Test for Antibacterial Activity

A preliminary screening test for testing the antibacterial activity of CDPG was performed. For this, all the bacterial strains used in the study were tested against a fixed concentration of 500 mg/mL of CDPG. Briefly, the bacterial strains were inoculated into BHIB containing 500 mg/mL of CDPG and incubated at 37 °C and 60 rpm in a microtiter plate for 48 h. Then, 10 µL of test inoculum was plated onto BHIA at 2 h, 4 h, 6 h, 24 h, and 48 h and incubated at 37 °C for 24 h. The growth or absence of bacterial growth on the BHIA plate was recorded.

#### 4.3.2. Detection of Minimum Inhibitory Concentration (MIC) of CDPG by the Microbroth Dilution Method

The MIC of the CDPG was evaluated according to Clinical and Laboratory Standards Institute guidelines (CLSI) by the microbroth dilution method [32]. Concentrations of CDPG ranging from 0.25 mg/mL to 500 mg/mL were prepared in BHIB by the microbroth double dilution method and tested against the bacterial strains (inoculum of 10^5^ CFU/mL). The results were recorded after an incubation period of 24 h at 37 °C. The lowest concentration of CDPG at which visible bacterial growth was not detected was identified as the minimum inhibitory concentration.

#### 4.3.3. Time-Kill Assay

A time-kill assay was performed as described previously [33]. Three concentrations of CDPG were tested; 500 mg/mL, 250 mg/mL, and 125 mg/mL against a fixed concentration of bacteria (10^5^ CFU/mL) [34]. Briefly, the bacterial strains were inoculated into BHIB containing different concentrations of CDPG and incubated for 24 h at 37 °C and 60 rpm in a microtiter plate. Bacterial strains inoculated and incubated for the same period, in the absence of CDPG, were used as a control set. Then, 10 µL of test inoculum were plated onto BHIA at 0 h, 2 h, 4 h, 6 h, 8 h, and 24 h and incubated at 37 °C for 24 h. Readings were recorded and colony forming units/mL (CFU/mL) were calculated. This was then converted to log10 values and compared with those values for the control (bacteria growing in absence of CDPG). The data were plotted as mean ± standard deviation as a function of time with a nonlinear regression using GraphPad Prism 5.

### 4.4. Antibiofilm Activity Using Crystal Violet Assay

In this study, 150 µL aliquots of bacteria in a BHIB suspension at a final concentration of 1.0 × 10^8^ CFU/mL were transported to a 96 well microplate and incubated at aerobic conditions for 24 h at 37 °C to allow for the biofilm formation [35]. After incubation, the microplate contents were emptied from each well and supplemented with 150 µL of fresh BHIB with CDPG and incubated at 37 °C for 24 h. CDPG was used at a concentration of 100 mg/mL, a concentration that is slightly smaller than the MIC, which was observed at 125 mg/mL. A control set without CPDG was also incubated. The wells were then washed gently thrice and were stained with 150 µL of 0.1% (wt/vol) crystal violet solution and incubated at room temperature for 15 min. Then, the wells were washed twice with phosphate buffer saline (PBS) and 150 µL of 95% ethanol was added to each well. The plates were incubated at room temperature for 10 min. The solution in each well was then transferred to a fresh microtiter plate, and the optical density (OD) was measured at 570 nm. The assay was done in triplicate, and the results were recorded as mean ±SD for both the test and the control.

#### Visualization of Biofilm Disruption by Confocal Laser Scanning Microscopy

Confocal laser scanning microscopy was carried out to confirm the results of crystal violet staining. Hydroxyapatite pellets of 3 mm^3^ were placed in each well of a 24-well microtiter plate. Then 1 mL of BHIB was added and inoculated with 5 µL of respective bacterial strain (0.5 McFarland). The samples were then incubated at 37 °C for 24 h for the formation of the biofilm. For each bacterial strain, there were two sets—control and test. On day 2, the used media was removed, and each well was supplemented with 1 mL of fresh BHIB. Only for the test wells, CDPG at a concentration of 100 mg/mL was added and incubated at 37 °C for 24 h at 60 rpm. The control wells on the other hand received no CDPG. On day 3, the hydroxyapatite pellets were carefully taken from the wells, placed on a 90 mm Petri dish (ABDOS Life Science, Roorkee, India), stained with DAPI (4′,6-diamidino-2-phenylindole, Abcam, UK), incubated at room temperature for 15 min in the dark, and observed under confocal laser scanning microscopy (CLSM, Nikon, Eclipse Ti, Japan). DAPI binds preferably to dsDNA and its blue fluorescence is proportional to the amount of the present DNA. Accordingly, DAPI was used to stain all cells in the biofilm [36].

### 4.5. Statistical Analysis

A one way analysis of variance (ANOVA) was used to test the significant difference between groups followed by *t*-tests to identify the pairwise difference among the groups. The statistical analysis was carried out using GraphPad Prism 5 software (San Diego, CA, USA) with a significance level of *p* value of ≤0.05.

## 5. Conclusions

According to the results of this study, significant antibacterial activity against all the tested bacteria was observed at 24 and 48 h of incubation with CDPG at a concentration of 250 mg/mL for the oral pathobionts and 500 mg/mL for multidrug-resistant pathogens. The average log10 CFU/mL reduction ranged from 2.66 to 9.65 according to the concentration of CDPG, time of treatment, and bacterial species. *L. casei* and *S. oralis* were completely killed by 250 mg/mL of CDPG after 24 h of treatment. *E. coli*, *E. fecalis, S. aureus,* and *P. aeruginosa*, were completely killed by 500 mg/mL of CDPG after 24 h of treatment. Furthermore, a significant reduction in biofilm formation was observed for all the tested bacteria grown in the presence of 100 mg/mL of CDPG. Accordingly, CDPG could be used as an antibacterial agent as well as an antibiofilm agent against oral biofilm-forming bacteria without the problem of developing antibiotic-resistant strains. However, further research on biocompatibility of the CDPG need to be carried out before the animal studies and human trials.

## Data Availability

Data available on request from the authors.

## Sample Availability

Samples of the compounds are not available from the authors.

## References

[1] Kasraei S., Sami L., Hendi S., Alikhani M.-Y., Rezaei-Soufi L., Khamverdi Z. (2014). Antibacterial properties of composite resins incorporating silver and zinc oxide nanoparticles on *Streptococcus mutans* and *Lactobacillus*. Restor. Dent. Endod..

[2] Alghamdi S. (2022). Isolation and identification of the oral bacteria and their characterization for bacteriocin production in the oral cavity. Saudi J. Biol. Sci..

[3] Tominari T., Sanada A., Ichimaru R., Matsumoto C., Hirata M., Itoh Y., Numabe Y., Miyaura C., Inada M. (2021). Gram-positive bacteria cell wall-derived lipoteichoic acid induces inflammatory alveolar bone loss through prostaglandin E production in osteoblasts. Sci. Rep..

[4] Le M.N.-T., Kayama S., Yoshikawa M., Hara T., Kashiyama S., Hisatsune J., Tsuruda K., Onodera M., Ohge H., Tsuga K. (2020). Oral colonisation by antimicrobial-resistant Gram-negative bacteria among long-term care facility residents: Prevalence, risk factors, and molecular epidemiology. Antimicrob. Resist. Infect. Control.

[5] Hojo K., Nagaoka S., Ohshima T., Maeda N. (2009). Bacterial interactions in dental biofilm development. J. Dent. Res..

[6] Kolenbrander P.E. (2000). Oral microbial communities: Biofilms, interactions, and genetic systems. Annu. Rev. Microbiol..

[7] Aruni A.W., Dou Y., Mishra A., Fletcher H.M. (2015). The Biofilm Community-Rebels with a Cause. Curr. Oral Health Rep..

[8] O’Toole G.A. (2011). Microtiter dish biofilm formation assay. J. Vis. Exp. JoVE.

[9] Fernandes G.L., Delbem A.C.B., do Amaral J.G., Gorup L.F., Fernandes R.A., de Souza Neto F.N., Souza J.A.S., Monteiro D.R., Hunt A.M.A., Camargo E.R. (2018). Nanosynthesis of Silver-Calcium Glycerophosphate: Promising Association against Oral Pathogens. Antibiotics.

[10] Jakubovics N.S., Goodman S.D., Mashburn-Warren L., Stafford G.P., Cieplik F. (2021). The dental plaque biofilm matrix. Periodontology 2000.

[11] Saafan A., Zaazou M.H., Sallam M.K., Mosallam O., El Danaf H.A. (2018). Assessment of Photodynamic Therapy and Nanoparticles Effects on Caries Models. Open Access Maced. J. Med. Sci..

[12] Cao W., Zhang Y., Wang X., Chen Y., Li Q., Xing X., Xiao Y., Peng X., Ye Z. (2017). Development of a novel resin-based dental material with dual biocidal modes and sustained release of Ag(+) ions based on photocurable core-shell AgBr/cationic polymer nanocomposites. J. Mater. Sci. Mater. Med..

[13] Abou Neel E.A., Pickup D.M., Valappil S.V., Newport R.J., Knowles J.C. (2009). Bioactive functional materials: A perspective on phosphate-based glasses. J. Mater. Chem..

[14] Abou Neel E.A., Ahmed I., Pratten J., Nazhat S.N., Knowles J.C. (2005). Characterisation of antibacterial copper releasing degradable phosphate glass fibres. Biomaterials.

[15] Abou Neel E.A., Hossain K.M.Z., Abuelenain D.A., Abuhaimed T., Ahmed I., Valappil S.P., Knowles J.C. (2021). Antibacterial effect of titanium dioxide-doped phosphate glass microspheres filled total-etch dental adhesive on *S. mutans* biofilm. Int. J. Adhes. Adhes..

[16] Abou Neel E.A., Roohpour N., Padidar B., Mordan N.J., Bozec L. (2021). Biomimetic dentin repair with a dual-analogue phosphate glass-polyacrylate paste: A proof-of-concept. Mater. Chem. Phys..

[17] Valappil S.P., Coombes M., Wright L., Owens G.J., Lynch R.J., Hope C.K., Higham S.M. (2012). Role of gallium and silver from phosphate-based glasses on in vitro dual species oral biofilm models of Porphyromonas gingivalis and *Streptococcus gordonii*. Acta Biomater..

[18] Abou Neel E.A., Kiani A., Valappil S.P., Mordan N.M., Baek S.-Y., Hossain K.M.Z., Felfel R.M., Ahmed I., Divakarl K., Chrzanowski W. (2019). Glass microparticle- versus microsphere-filled experimental dental adhesives. J. Appl. Polym. Sci..

[19] Abou Neel E.A., Young A.M., Nazhat S.N., Knowles J.C. (2007). A Facile synthesis route to prepare microtubes from phosphate glass fibres. Adv. Mater..

[20] Abou Neel E.A., Chrzanowski W., Knowles J.C. (2008). Effect of increasing titanium dioxide content on bulk and surface properties of phosphate-based glasses. Acta Biomater..

[21] Abou Neel E.A., Mizoguchi T., Ito M., Bitar M., Salih V., Knowles J.C. (2007). In vitro bioactivity and gene expression by cells cultured on titanium dioxide doped phosphate-based glasses. Biomaterials.

[22] Marovic D., Haugen H.J., Negovetic Mandic V., Par M., Zheng K., Tarle Z., Boccaccini A.R. (2021). Incorporation of copper-doped mesoporous bioactive glass nanospheres in experimental dental composites: Chemical and mechanical characterization. Materials.

[23] Angelé-Martínez C., Nguyen K.V.T., Ameer F.S., Anker J.N., Brumaghim J.L. (2017). Reactive oxygen species generation by copper(II) oxide nanoparticles determined by DNA damage assays and EPR spectroscopy. Nanotoxicology.

[24] Foroutan F., McGuire J., Gupta P., Nikolaou A., Kyffin B.A., Kelly N.L., Hanna J.V., Gutierrez-Merino J., Knowles J.C., Baek S.Y. (2019). Antibacterial Copper-Doped Calcium Phosphate Glasses for Bone Tissue Regeneration. ACS Biomater. Sci. Eng..

[25] Schroeder M., Brooks B.D., Brooks A.E. (2017). The complex relationship between virulence and antibiotic resistance. Genes.

[26] Yang Y.-I., Jung D.-W., Bai D.G., Yoo G.S., Choi J.-K. (2001). Counterion-dye staining method for DNA in agarose gels using crystal violet and methyl orange. Electrophoresis.

[27] Bonnekoh B., Wevers A., Jugert F., Merk H., Mahrle G. (1989). Colorimetric growth assay for epidermal cell cultures by their crystal violet binding capacity. Arch. Derm. Res..

[28] Colvin K.M., Gordon V.D., Murakami K., Borlee B.R., Wozniak D.J., Wong G.C., Parsek M.R. (2011). The pel polysaccharide can serve a structural and protective role in the biofilm matrix of Pseudomonas aeruginosa. PLoS Pathog..

[29] Hill C. (2012). Virulence or niche factors: What’s in a name?. J. Bacteriol..

[30] Chahal G., Quintana-Hayashi M.P., Gaytán M.O., Benktander J., Padra M., King S.J., Linden S.K. (2022). *Streptococcus oralis* Employs Multiple Mechanisms of Salivary Mucin Binding That Differ Between Strains. Front. Cell. Infect. Microbiol..

[31] Vestby L.K., Grønseth T., Simm R., Nesse L.L. (2020). Bacterial biofilm and its role in the pathogenesis of disease. Antibiotics.

[32] Weinstein M.P., Lewis J.S. (2020). 2nd. The Clinical and Laboratory Standards Institute Subcommittee on Antimicrobial Susceptibility Testing: Background, Organization, Functions, and Processes. J. Clin. Microbiol..

[33] Richard Schwalbe L.S.-M., Avery C.G. (2007). Methods for Determining Bactericidal Activity and Antimicrobial Interactions: Synergy Testing, Time-Kill Curves, and Population Analysis.

[34] Adusei E.B.A., Adosraku R.K., Oppong-Kyekyeku J., Amengor C.D.K., Jibira Y. (2019). Resistance Modulation Action, Time-Kill Kinetics Assay, and Inhibition of Biofilm Formation Effects of Plumbagin from *Plumbago zeylanica* Linn. J. Trop. Med..

[35] Coffey B.M., Anderson G.G. (2014). Biofilm formation in the 96-well microtiter plate. Methods Mol. Biol..

[36] Goodman S.D., Bakaletz L.O. (2022). Bacterial Biofilms Utilize an Underlying Extracellular DNA Matrix Structure That Can Be Targeted for Biofilm Resolution. Microorganisms.

